# Supplementary material for: Pan-cancer characterization of ncRNA synergistic competition uncovers potential carcinogenic biomarkers
Source: PLoS Comput Biol. 2023 Oct 9;19(10):e1011308. doi: 10.1371/journal.pcbi.1011308 (PMC10586676; doi:10.1371/journal.pcbi.1011308)
Supplement: Attachment — Submitted filename: Response to Reviewers.docx [file pcbi.1011308.s014.docx]

Dear Editor and Reviewers,

Thank you very much for your comments on our manuscript, entitled “Pan-cancer characterization of ncRNA synergistic competition uncovers potential carcinogenic biomarkers” (Manuscript ID: PCOMPBIOL-D-23-01031). All the comments and suggestions are very helpful for improving the quality of the paper.

We have revised the manuscript by following the comments carefully. Our responses to all the comments, including the details on how the manuscript has been revised, are provided in the following.

**Responses to Reviewers' Comments**

**Reviewer #1:**

**Comment R1.1:** Non-coding RNAs (ncRNAs) have emerged as significant regulators of gene expression and have been found to play crucial roles in the physiology and development of malignant tumors. The paper “Pan-cancer characterization of ncRNA synergistic competition uncovers potential carcinogenic biomarkers” provided a framework named as SCOM to show the synergistic competition of competing endogenous RNA (ceRNA), and further present the application of SCOM in drug resistance, molecular subtypes of malignant tumors, and immune regulation. A web-based database SCOMdb has been developed as part of this work, serving as a valuable resource for investigating ncRNA regulation. The manuscript addressed a relevant question in an emerging field of investigation, however, there are several limitations that hamper of the work.

**Response:** Thanks for the positive comment on the method/database developed by our paper and the suggestions. In this version, we have revised the paper by following all the comments carefully, as described below in the responses to your detailed comments.

**Comment R1.2:** The regulation of ncRNA-mRNA interaction needs the presence of transcription factors and miRNAs frequently. The authors should also check for the interaction among miRNA and TFs, and see if they could be involved in a more complex transcriptional circuit (ceRNAs+miRNAs+TFs+TF_targeting mRNAs).

**Response:** Thanks for the comment. Transcriptional regulation by transcription factors (TFs) and post-transcriptional regulation by miRNAs are often coordinated, so it is true that the ncRNA regulation needs the presence of TFs and miRNAs. In this work, we focus on studying ncRNA synergistic competition at the post-transcriptional level and hence only consider post-transcriptional regulation by miRNAs. However, it would be a promising direction to explore the interplay between TFs and miRNAs in the ceRNA regulation. In this version, we have clarified the focus of our study and point out this future direction in the “Conclusions and discussion” section as follows:

Here, we focus on studying ncRNA synergistic competition by only taking account of post-transcriptional regulation by miRNAs. However, in addition to miRNAs, transcription factors (TFs) also have a widespread effect on gene expression, and TFs and miRNAs are frequently coordinated to control the expression of their downstream genes [79]. In future, through considering both transcriptional regulation by TFs and post-transcriptional regulation by miRNAs, it is a promising direction to explore the interplay between TFs and miRNAs in the ceRNA regulation.

**Comment R1.3:** We always consider the expressions of ncRNAs and mRNAs, and the interaction networks are tissue or tumor specific. The study is pancancer characteristic. What do the authors think about this issue? How the consistency of molecular subtypes of malignant tumors and types of cancers? What is the application and significance of this framework in clinical experience?

**Response:** Thanks for the comment. We agree that for each tissue or tumor, the resulting interaction networks are tissue or tumor specific by considering the expressions of ncRNAs and mRNAs. However, research has shown that some interactions are conserved, i.e. they exist in more than one tissue or tumor networks; and some interactions are rewired, i.e. they exist in only one tissue or tumor network [1, 2]. Our study is still meaningful because we have revealed that malignant tumors tend to share hub ncRNAs rather than the ncRNA interactions involved in the synergistic competition.

Regarding your second question in the comment, there is still a lack of consensus on molecular subtypes, so the consistency of molecular subtypes of malignant tumors or cancers is hard to be evaluated. Currently, to obtain high-confidence results of molecular subtyping of malignant tumors, the common strategy is to use ensemble and consensus clustering methods [3]. Thus, in this work, we use an ensemble method implemented in the CancerSubtypes [4] R package (combining the Similarity Network Fusion (SNF) [5] and Consensus Clustering (CC) [6] methods) to generate malignant tumor subtypes. Moreover, for each malignant tumor, the number of molecular subtypes is empirically set according to the previous studies on molecular subtyping. In addition, we compute the silhouette width to measure how well a tumor sample is matched to its identified subtype compared to other subtypes, and a high value indicates that the tumor sample is well matched.

As for the application and significance of SCOM in clinical experience, we conduct a series of functional analyses including enrichment analysis, molecular subtyping analysis, multi-class classification analysis, survival analysis, and immune regulation analysis for the synergistic competition ncRNAs identified by using SCOM. We have found that the conserved ncRNA synergistic competition ncRNAs and hub ncRNAs are significantly enriched in diseases, cell markers and cancer hallmarks. In addition, the conserved and rewired hub ncRNAs may act as potential diagnostic and prognostic biomarkers across malignant tumors. Moreover, the synergistic competition ncRNAs are likely to be involved in drug resistance, contribute to distinguishing molecular subtypes of malignant tumors, and participate in immune regulation. These results indicate that the SCOM framework can effectively infer the synergistic competition ncRNAs with biological significance. Although experimentally validated carcinogenic biomarkers are limited, we have discovered that a significant number of synergistic competition ncRNAs are confirmed as carcinogenic biomarkers by the literature (Fig S8 in S1 File, S12 Data). Altogether, SCOM can be used as a tool for studying ncRNAs and identifying potential carcinogenic markers.

References

[1] Xu J, Li Y, Lu J, Pan T, Ding N, Wang Z, et al. The mRNA related ceRNA-ceRNA landscape and significance across 20 major cancer types. Nucleic Acids Res. 2015;43(17):8169-82. doi: 10.1093/nar/gkv853.

[2] Xu J, Feng L, Han Z, Li Y, Wu A, Shao T, et al. Extensive ceRNA-ceRNA interaction networks mediated by miRNAs regulate development in multiple rhesus tissues. Nucleic Acids Res. 2016;44(19):9438-9451. doi: 10.1093/nar/gkw587.

[3] Zhao L, Lee VHF, Ng MK, Yan H, Bijlsma MF. Molecular subtyping of cancer: current status and moving toward clinical applications. Brief Bioinform. 2019;20: 572–584. doi:10.1093/bib/bby026

[4] Xu T, Le TD, Liu L, Su N, Wang R, Sun B, et al. CancerSubtypes: an R/Bioconductor package for molecular cancer subtype identification, validation and visualization. Bioinformatics. 2017;33: 3131–3133. doi:10.1093/bioinformatics/btx378

[5] Wang B, Mezlini AM, Demir F, Fiume M, Tu Z, Brudno M, et al. Similarity network fusion for aggregating data types on a genomic scale. Nat Methods. 2014;11: 333–337. doi:10.1038/nmeth.2810

[6] Monti S, Tamayo P, Mesirov J, Golub T. Consensus clustering: a resampling-based method for class discovery and visualization of gene expression microarray data. Machine Learning. 2003;52: 91–118. doi:10.1023/A:1023949509487

**Comment R1.4:** In the method to recruit ncRNA, the criterion requires that ncRNA i and ncRNA j are positively correlated. How about the negatively correlated ncRNAs?

**Response:** Thanks for the comment. Since negative correlation is a relationship between two ncRNAs in which one ncRNA increases as the other ncRNA decreases, the negatively correlated ncRNA-ncRNA pairs are not likely to be synergistic with each other. So, in this work, the negatively correlated ncRNA-ncRNA pairs are not considered as candidate synergistic competition pairs, so they are removed.

**Comment R1.5:** The authors used the Pan-cancer transcriptomics data including the expression data of ncRNAs (including miRNAs, lncRNAs and pseudogenes) and mRNA. An important point remains to be highlighted that the stoichiometric role of these molecules. This point is referred to as the equimolarity assumption which leads to the prediction of potential ceRNA competition effect around the molecular equilibrium.

**Response:** Thanks for the advice. Yes, the stoichiometry of miRNAs and ncRNAs is a vital factor to predict ceRNA interactions. As suggested, in this version, we have highlighted the stoichiometry of miRNAs and ncRNAs when predicting ceRNA interactions of a ncRNA of interest in the section “Conclusions and discussion” as follows:

The prediction of ncRNA-related ceRNA network is a critical step to infer ncRNA synergistic competition. Yet, there is still a lack of study on the prediction of ncRNA-related ceRNA networks. In the current work, we apply three criteria (significant sharing of miRNAs, significant positive correlation, and significant sensitive correlation conditioning on shared miRNAs) to predict ncRNA-related ceRNA networks in the 31 malignant tumors. Since the three criteria have been successfully used to investigate ceRNA regulation [27,35,71–73], the ncRNA-related ceRNA networks predicted by SCOM are promising. However, SCOM doesn’t consider the stoichiometry of miRNAs and ncRNAs when predicting ceRNA networks. Since miRNA efficacy is determined by its cellular abundance [74] or its abundance within RNA-induced silencing complexes (RISC) [75], the stoichiometry of miRNAs and ncRNAs is considered as a vital factor to predict ceRNA interactions of a ncRNA of interest [76]. An implication of understanding the stoichiometry of miRNAs and ncRNAs is that ncRNAs with higher expression levels are more likely to have greater competitive ability [10]. Therefore, to accurately predict ncRNA-related ceRNA interactions, SCOM will select highly expressed ncRNAs across malignant tumors in future. With deeper understanding of ceRNA mechanisms, we can more accurately predict ncRNA-related ceRNA networks to improve the inference of ncRNA synergistic competition and accelerate the discovery of carcinogenic biomarkers.

**Reviewer #2:**

**Comment R2.1:** In their manuscript "Pan-cancer characterization of ncRNA synergistic competition uncovers potential carcinogenic biomarkers", Junpeng Zhang et al. describe a new method SCOM which focuses on unraveling synergistic competition in competing endogenous RNA (ceRNA) networks. Following the hypothesis of ceRNA by Salmena et al., transcripts carrying microRNA binding sites compete for a limited pool of microRNA, affecting each other through de-repression. This effect is investigated here from a new perspective of synergy between ceRNAs, where multiple ceRNAs may boost each other's effect on the network by their joint activity. ceRNAs in synergistic competition are in SCOM those non-coding RNAs that show a significant overlap of mRNA targets (hypergeometric test), have significant positive correlation themselves and both show significant sensitivity correlation considering shared mRNA targets. Notably, sensitivity correlation is used here to first infer the ceRNA network (as it was used before by Paci et al. (2014) and List et al. (2019)) and then again to investigate the competition of two non-coding RNAs conditioning on shared mRNAs. The method is described in sufficient details to allow reproducing it. The results are comprehensive and deeply characterized from multiple angles to help readers understand the meaning of the resulting networks.

**Response:** Thanks for the summary comment. In this version, we have revised the paper by following all the comments carefully, and please find below our responses to your detailed comments.

**Comment R2.2:** I welcome the idea to look for synergies between ceRNAs in co-regulating the network. However, by using the concept of sensitivity correlation twice, once for establishing the shared ceRNA network and again, by studying competition of pairs of non-coding RNAs, I wonder if this represents a case of double-dipping as the correlation coefficients considered here are included in both tests. To avoid this, one should split the data and use part to infer the ceRNA network while using the other part to study synergistic competition.

**Response:** Thanks for the comment. As the reviewer said, the concept of sensitivity correlation is used twice, but it is used for two different scenarios: ceRNA network construction and ncRNA synergistic competition network construction. For the ceRNA network construction, the sensitivity correlation test is done for a ncRNA-mRNA pair, i.e. the test is used to evaluate the influence of the shared miRNAs on a ncRNA and an mRNA. For the ncRNA synergistic competition network construction, the sensitivity correlation test is done for a ncRNA pair, i.e. the test is used to evaluate the influence of synergistically competed mRNAs on two different ncRNAs. Since the sensitivity correlation tests are used for obtaining two different types of interactions (ncRNA-mRNA and ncRNA-ncRNA), we would not consider this is a case of double-dipping.

In addition, SCOM is a step-wise framework rather than a train-test method to study ncRNA synergistic competition, and the ncRNA synergistic competition network construction (the second step) depends on the identified ceRNA networks (the first step) in the same dataset. So we do not split the data into two parts such that one part to infer the ceRNA network and the other part to study the ncRNA synergistic competition network.

**Comment R2.3:** It is not clear why the authors suspect synergistic competition only between pairs of non-coding RNAs. Pairs of mRNAs and mixed pairs of non-coding/coding RNAs quite likely also exhibit this behavior, especially given that the most powerful ceRNAs in a network are often mRNAs (as shown in the SPONGE paper, List et al. 2019). I can imagine that the authors opted for this split to keep a clear separation between regulatory (non-coding RNAs) and regulated genes (mRNAs) but since all transcripts can in principle fill both roles, this limitation of the current study should be discussed critically.

**Response:** Thanks for pointing out this limitation. Yes, pairs of mRNAs and mixed pairs of non-coding/coding RNAs also show the synergistic competition. To uncover the roles of ncRNAs acting as ceRNAs in terms of the synergistic competition between them, we focus on the synergistic competition between pairs of ncRNAs in this work. In this version, we have discussed the limitation in the section “Conclusions and discussion” as follows:

In this work, SCOM is focused on studying the synergistic competition between pairs of ncRNAs. However, mRNAs, when acting as ceRNAs, are also important members in ceRNA networks. Therefore, the pairs of mRNAs and the mixed pairs of ncRNAs/mRNAs are also likely to synergistically compete with miRNA targets. In the future, we will extend SCOM for investigating the synergistic competition between the pairs of mRNAs and the mixed pairs of ncRNAs/mRNAs as well.

**Comment R2.4:** In the conclusion, the authors mention the importance of stoichiometry for the effectiveness of ceRNA regulation. A more basic step that I would have expected here is to check for the expression of the synergistic ceRNAs - if those expression values are very low, they are not likely to have a substantial effect; it may thus be better to focus on the more highly expressed examples or to use weighted approaches in the interpretation/enrichment analyses.

**Response:** Thanks for the comment. Since miRNA efficacy is determined by its cellular abundance or its abundance within RNA-induced silencing complexes (RISC), it is true that ceRNAs with higher expression values are more likely to have greater competitive ability. In this version, we have improved the statement in the section “Conclusions and discussion” as follows.

The prediction of ncRNA-related ceRNA network is a critical step to infer ncRNA synergistic competition. Yet, there is still a lack of study on the prediction of ncRNA-related ceRNA networks. In the current work, we apply three criteria (significant sharing of miRNAs, significant positive correlation, and significant sensitive correlation conditioning on shared miRNAs) to predict ncRNA-related ceRNA networks in the 31 malignant tumors. Since the three criteria have been successfully used to investigate ceRNA regulation [27,35,71–73], the ncRNA-related ceRNA networks predicted by SCOM are promising. However, SCOM doesn’t consider the stoichiometry of miRNAs and ncRNAs when predicting ceRNA networks. Since miRNA efficacy is determined by its cellular abundance [74] or its abundance within RNA-induced silencing complexes (RISC) [75], the stoichiometry of miRNAs and ncRNAs is considered as a vital factor to predict ceRNA interactions of a ncRNA of interest [76]. An implication of understanding the stoichiometry of miRNAs and ncRNAs is that ncRNAs with higher expression levels are more likely to have greater competitive ability [10]. Therefore, to accurately predict ncRNA-related ceRNA interactions, SCOM will select highly expressed ncRNAs across malignant tumors in future. With deeper understanding of ceRNA mechanisms, we can more accurately predict ncRNA-related ceRNA networks to improve the inference of ncRNA synergistic competition and accelerate the discovery of carcinogenic biomarkers.

**Comment R2.5:** The authors use the pt function (t distribution) to estimate if the Pearson correlation coefficient is significant. This might not be accurate as the independence assumptions are likely violated. The authors could account for this by obtaining an empirical p-value based on a null distribution of correlation coefficients obtained from gene pairs not sharing miRNAs.

**Response:** Thanks for the comment. It is a good way to obtain empirical *p*-values of Pearson correlation coefficient based on a null distribution of correlation coefficients obtained from gene pairs not sharing miRNAs, but it is usually computationally intensive. Although there is an independence assumption in the *pt* function, the function (Student t-distribution) is still a common and efficient method to approximately estimate if the Pearson correlation coefficient is significant. Therefore, in this work, we use *pt* function to approximately calculate the significance of Pearson correlation coefficient.

**Comment R2.6:** If I understood correctly, the power law property is investigated by comparing the obtained SCOM network against random networks using a KS test on the path lengths. To my knowledge, this is unusual. A more common way to assess the power law property is to check if a power law can be fitted to the lower part of the node degree distribution as done by the poweRlaw R package, for example.

**Response:** Thanks for the comment. For the power law property, we don’t compare the obtained ncRNA synergistic competition networks against their corresponding random networks using Kolmogorov-Smirnov (KS) test. The KS test is directly used to evaluate whether the node degree of a ncRNA synergistic competition network follows a power law distribution.

To assess the power law property, the poweRlaw R package is commonly used. Meanwhile, other tools, such as igraph is often used too. It is noted that igraph and poweRlaw all use the Kolmogorov-Smirnov statistic to minimise the distance between the data and the fitted model cumulative density function (CDF). For igraph and poweRlaw, their implementation principles of fitting power-law distribution are the same (both are based on the literature “Aaron Clauset, Cosma R .Shalizi and Mark E.J. Newman: Power-law distributions in empirical data. SIAM Review 51(4):661-703, 2009.”). Moreover, for network object, the igraph package is more convenient and popular to conduct network topological analysis, so we apply it to assess the power law property.

**Comment R2.7:** The (multi-class) classification performance was compared against a baseline method but not against a random baseline - please repeat the classification after sample label (subtype label) permutation to see what a random baseline would look like.

**Response:** Thanks for the advice. In this version, we have added a comparison in terms of multi-class classification performance with a random prediction method implemented in the R package utiml. We have found that the conserved ncRNA-related ceRNA regulations, the conserved hub ncRNAs, and the rewired hub ncRNAs identified by SCOM have better performance than the baseline method and the random prediction method in classifying the 31 malignant tumors (Fig S5 in S1 File, Fig 4c, and Fig S7c in S1 File). Moreover, the synergistic competition ncRNAs identified by SCOM have better performance than the baseline method and the random prediction method in classifying BRCA subtypes (Fig 6d).

**Comment R2.8:** The classification results would be more convincing if applied to another data set, e.g. METABRIC for breast cancer.

**Response:** Thanks for the suggestion. In this version, we have added another different BRCA dataset from METABRIC. By applying multi-class classification analysis to the BRCA dataset from METABRIC, we have shown that the synergistic competition ncRNAs also have better performance than the baseline method and the random prediction method in classifying BRCA subtypes (Table S1 in S1 File).

**Comment R2.9:** SCOMdb makes the results of this study easily accessible to users. However, the manuscript should also mention the state of the art, since there are numerous other web resources like SPONGEdb that SCOMdb should be compared against. https://doi.org/10.1002/wrna.1686 offers a review in Table 1 of such resources.

**Response:** Thanks for the advice. In this version, we have mentioned the other web resources and the main difference between SCOM and them in the section “SCOMdb: a web-based resource for ncRNA synergistic competition in Pan-cancer” as follows:

Different from the existing databases of collecting ceRNA interactions (e.g. ENCORI [33], SPONGEdb [65], LncACTdb [66], and LnCeCell [67], see [68] for a summary of databases for ceRNAs), SCOMdb concentrates on providing the synergistic competition interactions of ncRNAs acting as ceRNAs. The provided resources of SCOM will be continuously updated, and can serve as a valuable resource for biologists interested in investigating ncRNA synergistic competition across malignant tumors.

**Comment R2.10:** The R code is offered as a download on the SCOMdb site but it would be much more convenient if the GitHub address was listed. The authors should also turn their code into an R package so it can be installed directly from GitHub via devtools.

**Response:** Thanks for the comment. In this version, we have listed the GitHub address (https://github.com/zhangjunpeng411/SCOM/) in the “Abstract”. Moreover, the code of SCOM has been turned into an R package, which is available at the GitHub site.

**Comment R2.11:** The methods section is partially repetitive, e.g. the three criteria for SCOM are repeated several times.

**Response:** Thanks for the comment. In this version, we have improved the presentation by minimizing the repetitions in the section “Materials and methods”. For example, we improved the repetitive contents of “8001 matched tumor samples” and “three criteria”.

**Comment R2.12:** Page 12, bottom "when the influence of the synergistically competed mRNAs is eliminated". You mean miRNA here, not mRNA.

**Response:** Thanks for the comment. We mean mRNA rather than miRNA in the sentence “when the influence of the synergistically competed mRNAs is eliminated”. This is because we aim to evaluate the influence of synergistically competed mRNAs on ncRNA *i* and ncRNA *j*.

**Comment R2.13:** The authors found the number of ceRNA interactions of pseudogene-related ceRNA networks to be significantly larger than that of lncRNA-related ceRNA networks. Not so surprising given that there are much more pseudogenes than lncRNA considered.

**Response:** Thanks for the comment. We have revised the statement into “Generally, the number of ceRNA interactions of the pseudogene-related ceRNA networks is significantly larger than that of the lncRNA-related ceRNA networks (Fig 2a, *p*-value = 1.84E-13 with paired Student’s *t*-test). This result may be explained by the fact that the number of pseudogenes is larger than that of lncRNAs in the transcriptomics data.”.

**Comment R2.14:** It is not getting clear why the power law behavior is really important to consider. What does this change and mean?

**Response:** Thanks for the comment. Generally, the degree distributions of biological networks are power law in the real world. Since being scale-free is a property of a complex network (e.g. a biological network) and the node degree of a scale-free biological network follows a power law distribution, in this study, we consider the power law behavior to understand whether the ncRNA-related ceRNA networks and ncRNA synergistic competition networks tend to be scale-free networks and thus help us understand the underlying mechanism (e.g. preferential attachment) of these networks. In this version, we have clarified the importance of the power law distribution in the section “Network topological analysis” as follows:

Understanding the topological properties of biological networks, such as node degree, paths and network density can lead to useful insights into the networks. Being scale-free is a property of a complex network (e.g. biological network), and it is determined by the underlying mechanism (e.g. preferential attachment) of a biological network [36]. If the node degree of a biological network obeys a power law distribution, the biological network is regarded as a scale-free network, and this is an important feature of biological networks in the real world [37].

**Comment R2.15:** The authors might want to check out spongEffects in the future (https://doi.org/10.1093/bioinformatics/btad276). This method currently extracts modules from the ceRNA network by considering high-degree ceRNAs and their targets. SCOM delivers an alternative definition of a ceRNA module (a synergistic one) and its behavior across individual samples or subtypes might be better represented by spongEffect scores.

**Response:** Thanks for the recommendation. In this version, we have mentioned the spongeEffect scores (i.e. enrichment scores) in the section “Conclusions and discussion” as follows:

For multi-class classification analysis, we use the expression data of ceRNAs as input to train a classification model. Previous studies [77,78] have shown that using module (i.e. a set of ceRNAs) enrichment scores inferred from gene expression data can help to understand the regulatory activity of ceRNA modules, and is a promising way to classify tumor types or subtypes. As a future work, we plan to incorporate the enrichment scores of ceRNA modules with spongEffects [78] to SCOM, and further them as part of the input to train a classification model when classifying tumor types or subtypes.

**Comment R2.16:** The SCOMdb site is not accessible without the www prefix and does not use a secure connection. Both should be fixed.

**Response:** Thanks for the advice. Both have been fixed.

**Comment R2.17:** In SCOMdb, the search window is currently also a bit poor in features, e.g. result tables can not be sorted, filtered or downloaded. There are no visualizations (like bar charts, box or violin plots etc.) to illustrate the results. This should be improved in the future if the authors intend for their users to benefit from this page.

**Response:** Thanks for the advice. In this version, we have further improved the database SCOMdb (https://comblab.cn/SCOMdb/).

**Reviewer #3:**

**Comment R3.1:** Due to its innovative creativity and well writing, I am willing to recommend that this manuscript be accepted and published. However, there are two issues that need to be explained in more detail by the author in the discussion.

**Response:** Thanks for the positive comment and the suggestions. In this version, we have revised the paper by following all the comments carefully, and please find below our responses to your comments.

**Comment R3.2:** Can the experimental path for verifying this theory be proposed in more detail in the discussion, rather than simply stating that further "experimental verification" is needed.

**Response:** Thanks for the advice. In this version, we provide an experimental path for verifying our theory in the section “Conclusions and discussion” as follows:

It is meaningful to design appropriate wet-lab experiments to confirm the predicted ncRNA synergistic competition relationships. For biological experiments, biologists can choose some ncRNAs of interest for validation. For example, to confirm whether *PVT1* and *H19* have synergistic competition relationships or not, biologists can develop an experimental framework to compare the suppressive effects of *PVT1* and *H19* on miRNA activity before and after knocking out one of them. In the two knockout experiments (one for *PVT1* and one for *H19*), if we can observe that several miRNAs show relatively stronger activity, *PVT1* and *H19* have synergistic competition relationships.

**Comment R3.3:** Regardless of the differences in tumor types (such as lung cancer, colorectal cancer, and/or pancreatic cancer), all types of tumor cells have common characteristics. Does the same SCOM exist in the systematic analysis at the pan cancer level in this study?

**Response:** Thanks for the comment. Yes, all types of tumors also show some common characteristics in our study. For example, in the section “The ceRNA regulation landscape in Pan-cancer”, our result shows that the malignant tumors may have common ncRNA-related ceRNA regulation. Moreover, in the section “The ncRNA synergistic competition landscape in Pan-cancer”, our result also indicates the malignant tumors may share a small portion of ncRNA synergistic competition with each other.

We would like to thank the editor and all reviewers again for your comments, which have greatly helped us to improve the quality of the paper.

Yours sincerely,

Junpeng Zhang & co-authors
